# Comparison of PERCIST5, imPERCIST5, and PERCIMT Criteria for Early Assessment of Pembrolizumab Response with FDG-PET/CT in Metastatic Bladder Cancer Patients

**DOI:** 10.3390/ph18050701

**Published:** 2025-05-09

**Authors:** Marc Bertaux, Caroline Luo, Camelia Radulescu, Philippe Beuzeboc, Cecile Landais, Pauline Touche, Christine Abraham, Marie Homo Seban, Eve Camps, Antoine Faucheron, Morgan Tourne, Lucie Fricot, Lea Turpin, Romain-David Seban, Sabrina Khedairia

**Affiliations:** 1 Department of Nuclear Medicine, Foch Hospital, 92100 Suresnes, France; luo.caroline1@gmail.com (C.L.); marie.homo@gmail.com (M.H.S.); l.turpin@hopital-foch.com (L.T.); 2 Department of Anatomopathology, Foch Hospital, 92100 Suresnes, France; cm.radulescu@hopital-foch.com (C.R.); m.tourne@hopital-foch.com (M.T.); lucie.fricot@mailo.com (L.F.); 3 Department of Oncology, Foch Hospital, 92100 Suresnes, France; p.beuzeboc@hopital-foch.com (P.B.); c.abraham@hopital-foch.com (C.A.); 4 Department of Clinical Research and Innovation, Foch Hospital, 92100 Suresnes, France; c.landais@hopital-foch.com (C.L.); p.touche@hopital-foch.com (P.T.); 5 Department of Pharmacy, Foch Hospital, 92100 Suresnes, France; e.camps@hopital-foch.com (E.C.); a.faucheron@hopital-foch.com (A.F.); sabrinasabik@gmail.com (S.K.); 6 Department of Nuclear Medicine, Institut Curie, 92210 Saint-Cloud, France; romaindavid.seban@curie.fr

**Keywords:** bladder cancer, immunotherapy, [18F]-fluorodeoxyglucose, positron emission tomography, Pembrolizumab

## Abstract

**Background/Objectives:** Immunotherapy is an essential part of metastatic bladder cancer treatment. Our main objective was to study the prognostic value of FDG-PET/CT in early assessment of response to Pembrolizumab in metastatic bladder cancers using PERCIST5, imPERCIST5, and PERCIMT criteria. **Methods**: A total of 42 patients were evaluated with FDG-PET/CT at baseline and after 3–4 cycles of Pembrolizumab. Treatment response was blindly assessed with PERCIST5, imPERCIST5, and PERCIMT. Imaging and clinical data were collected. Progression was defined clinically using oncologist reports. **Results**: A total of 37 patients were evaluable with the PERCIST5 and imPERCIST5 criteria and included in the analysis. Median disease-specific progression-free survival (PFS) and overall survival (OS) were 152 and 363 days, respectively. All response criteria were significantly associated with PFS. When response was dichotomized in responders versus non-responders all scores were significantly associated with OS. When response was dichotomized in progressors versus non-progressors, only PERCIST5 (hazard ratio (HR) 2.2) and PERCIMT (HR 2.6) were significantly associated with OS, while imPERCIST was not (HR 1.6). Two patients had pseudoprogression (5%), both being adequately classified as non-progressors with PERCIMT criteria. **Conclusions**: Early response to immunotherapy as assessed with FDG-PET is a strong prognostic factor in bladder cancer patients, especially using the PERCIST5 or PERCIMT criteria. The latter seems clinically useful as it is simple to perform and its specific definition of metabolic progression correctly ruled-out patients with significant clinical benefit of Pembrolizumab in our study.

## 1. Introduction

Bladder cancer ranks 9th among cancers worldwide in terms of incidence, and ranks 13th in terms of mortality, with 610,000 new cases diagnosed globally and 220,000 related deaths per year [1]. Approximately 25% of patients have muscle invasive cancer, which has a high rate of metastasis and a poor prognosis [2]. The treatment of metastatic bladder cancer was solely based on chemotherapy for a long time, with cisplatin-based regimen as first-line therapy for patients who could tolerate it. Immune checkpoint inhibitors (ICIs) targeting the PD-1/PDL-1 pathway were introduced in the treatment of bladder cancer in 2016–2017. Pembrolizumab demonstrated a survival benefit as second-line therapy (KEYNOTE-045) in this setting, leading to its approval as a preferred option for metastatic patients who progressed after platinum-based chemotherapy [3]. It represented a paradigm shift, offering durable responses in a subset of patients with metastatic cancer. However, challenges remain in identifying which patients will significantly benefit from immunotherapy, as only about 20–30% of patients with bladder cancer respond to these treatments, while they can be costly, and many patients probably would benefit from quick discontinuation of immunotherapy when it is ineffective. Nevertheless, evaluation of therapeutic response presents unique challenges due to immunotherapy distinct mechanism of action compared to cytotoxic chemotherapy. In particular, pseudoprogression can occur, where tumors initially appear to grow before regressing, complicating the use of conventional imaging criteria. These atypical response patterns led to the development and application of specialized criteria, such as iRECIST, used in computed tomography (CT) to assess immunotherapy efficacy [4]. The latter requires confirmation of progression on a follow-up scan before treatment is stopped. Alternatively, patients can be followed with [18F]-fluorodeoxyglucose (FDG) positron emission tomography coupled to CT (FDG-PET/CT). FDG-PET/CT first proved its clinical utility in the evaluation of immunotherapy response in patients with melanoma [5], and more recently in patients with lung cancer [6]. Most bladder cancers are FDG-avid, and FDG-PET/CT showed good performances in their initial staging [7], as well as potential usefulness in the evaluation of patients treated with neoadjuvant chemotherapy [8] or immunotherapy [9] for a localized tumor. However, data on its performance for the evaluation of metastatic patients treated with immunotherapy are lacking. Atypical response pattern of tumors to immunotherapy is also a challenge in metabolic imaging [10]. As a result, some alternative criteria to the most widely used FDG-PET response assessment score PERCIST have been specifically designed for evaluating patients treated with immunotherapy. In particular, imPERCIST5 and PERCIMT criteria were shown to differentiate patients treated for a metastatic melanoma with Ipilimumab according to their long-term prognosis [5,11,12], while PERCIST was not. The main objective of our study was to compare the prognostic values of imPERCIST5, PERCIMT, and the variation with maximum five target lesions of the more traditional response score PERCIST 1.0 (PERCIST5), in the early assessment of treatment response of metastatic bladder cancer patients treated with Pembrolizumab [13]. The secondary objectives were the description of response rate, types, and immune-related adverse events (irAEs) in this population. The article also includes a discussion on novel radiopharmaceuticals that are currently being studied in bladder cancer imaging.

## 2. Results

### 2.1. Patient Characteristics

**Figure 1:**
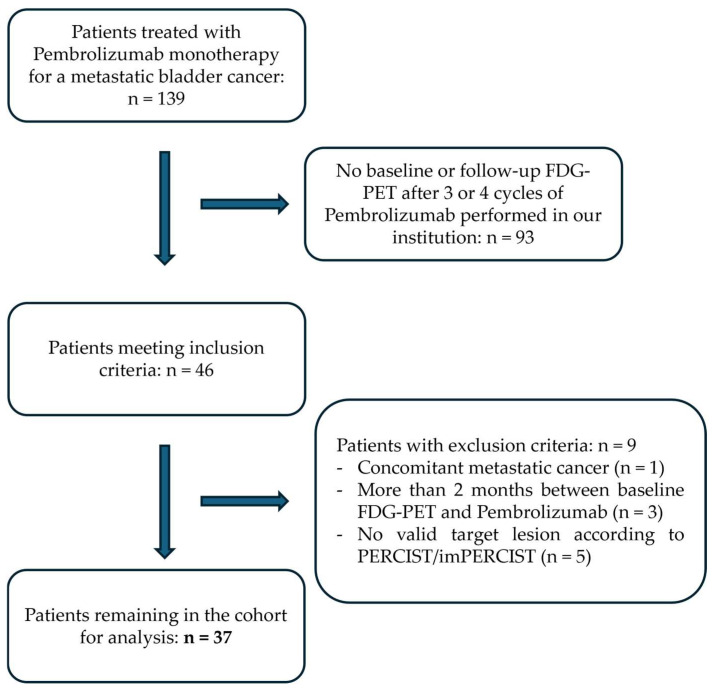
Study flow chart FDG-PET, [18F]-fluorodeoxyglucose positron emission tomography.

A total of 139 patients were scheduled for treatment of metastatic bladder cancer with Pembrolizumab monotherapy in our institution (Foch Hospital, Suresnes, 92150, France) between 12 December 2017 and 18 December 2022. Among them, 46 patients met the inclusion criteria with both baseline and follow-up FDG-PET/CT performed in our center. Nine patients were excluded from the study: three because more than two months had passed between baseline examination and first cycle of Pembrolizumab, one because of a concomitant metastatic prostate cancer, and five because they had no hypermetabolic lesion reaching the PERCIST/imPERCIST threshold to be considered a valid target on the FDG/PET-CT baseline scan. A total of 37 patients remained in the cohort for analysis, including 26 men (70%) and 11 women (30%), with a median age of 72 years, and a mean body weight of 72.1 (±13.9) kg. A flow chart of patient selection is presented in Figure 1. Nine patients had an Eastern Cooperative Oncology Group (ECOG) performance status score of 0 (24%), 22 had a score of 1 (60%), and 6 had a score of 2 (16%). A total of 25 patients received Pembrolizumab as second line of systemic treatment (68%), 10 patients as a third line (27%), and 2 patients as a fourth line (5%). Histological type of bladder cancer was reassessed in 33 patients. Classic urothelial carcinoma histology was found in 26 patients (79%) whereas variant urothelial carcinoma was found in 6 patients (18%, 2 micropapillary, 2 plasmacytoid, and 2 basal-like variants) and adenocarcinoma was found in one patient (3%). PDL-1 status was available for 25 patients (68%). Median tumor proportion score (TPS) and combined positive score (CPS) were 5% and 10%, respectively. Respectively, 11 (44%) and 9 patients (36%) had TPS and CPS scores < 1%. A difference in liver mean standard uptake value (SULmean) of more than 0.3 or 20% between baseline and evaluation FDG-PET/CT was seen in seven patients (19%). A difference in uptake time of more than 15 min was seen in four patients (11%). These differences were considered of minor importance, and did not preclude evaluation with the PERCIST or imPERCIST criteria. Median and mean disease-specific progression-free survival (PFS) were 152 (±29) days and 361 (±70) days, respectively, with nine patients (24%) having PFS > 1 year. Median and mean overall survival (OS) were, respectively, 363 (±47) days and 517 (±70) days, with nine patients (24%) having OS > 2 years. Patient characteristics are presented in Table 1. 

### 2.2. PERCIST5

Of the 37 included in the study, 5 (13.5%) had complete metabolic response (CMR), 5 (13.5%) had partial metabolic response (PMR), 5 (13.5%) had stable disease (SD), and 22 (59.5%) had progressive metabolic disease (PMD). Grouping CMR + PMR versus SD + PD (i.e., responders versus non-responders) resulted in best performances to predict PFS < 1 year, with sensitivity of 85.7% and specificity of 66.7%. PFS was significantly longer (*p* = 0.001) in responders (median PFS not reached) than in non-responders (median PFS 136 ± 18 days), with a hazard ratio (HR) of 5.57 (1.88–16.51). Grouping CMR + PMR + SD versus PMD (i.e., non-progressors versus progressors) resulted in sensitivity of 71.4% and specificity of 77.8% to predict PFS < 1 year. PFS was significantly longer (*p* < 0.001) in non-progressors (median PFS 754 ± 399 days) than in progressors (median PFS 109 ± 30 days), with a HR of 5.51 (2.29–13.29).

**Figure 2:**
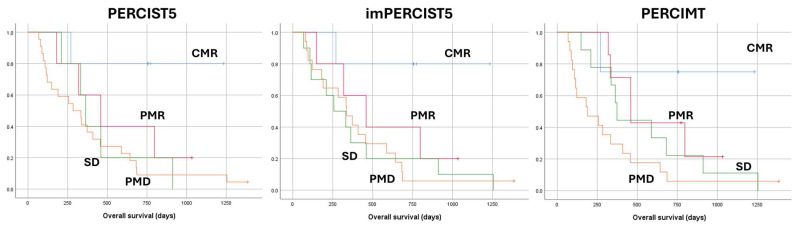
Kaplan–Meier overall survival curves according to the PERCIST5, imPERCIST5, and PERCMT criteria. Complete metabolic response (CMR) is associated with the longest overall survival using all scores. While PERCIMT appears to better distinguish prognosis between patients with stable disease (SD) and progressive metabolic disease (PMD), this difference is not statistically significant (*p* = 0.21). The prognostic of patients with partial metabolic response (PMR) is closer to that of patients with SD than it is to that of patients with CMR.

Grouping responders versus non-responders resulted in the best performances to predict OS < 2 years, with sensitivity of 85.7% and specificity of 66.7%. OS was significantly longer (*p* = 0.016) in responders (median OS 797 ± 327 days) than in non-responders (334 ± 46 days), with a HR of 3.11 (1.18–8.19). Grouping non-progressors versus progressors resulted in sensitivity of 71.4% and specificity of 77.8% to predict OS < 2 years. OS was significantly longer (*p* < 0.037) in non-progressors (median OS 460 ± 224 days) than in progressors (284 ± 86 days), with HR of 2.22 (1.03–4.78). HR of Cox survival analyses are presented in Table 2. Survival curves are presented in Figure 2.

### 2.3. ImPERCIST5

Of the 37 included in the study, 5 had CMR (13.5%), 5 had PMR (13.5%), 10 had SD (27%), and 17 had PD (46%). Grouping responders versus non-responders resulted in the best performances to predict PFS < 1 year, with sensitivity of 85.7% and specificity of 66.7%. PFS was significantly longer (*p* = 0.001) in responders (median PFS not reached) than in non-responders (median PFS 140 ± 19 days), with HR of 5.36 (1.80–15.92). Grouping non-progressors versus progressors resulted in sensitivity of 57.1% and specificity of 88.9%. PFS was significantly longer (*p* < 0.001) in non-progressors (median PFS 282 ± 62 days) than in progressors (median PFS 119 ± 42 days), with HR of 3.73 (1.68–8.26).

Grouping responders versus non-responders resulted in best performances to predict OS < 2 years, with sensitivity of 85.7% and specificity of 66.7%. OS was significantly longer (*p* = 0.017) in responders (median OS 797 ± 327) than in non-responders (334 ± 46 days), with HR of 3.09 (1.17–8.15). Grouping non-progressors versus progressors resulted in sensitivity of 57.1% and specificity of 88.9%. OS was not significantly different (*p* = 0.18) between non-progressors (median OS 363 ± 142 days) and progressors (337 ± 61 days), with HR of 1.63 (0.79–3.33).

### 2.4. PERCIMT

On the 37 included in the study, 4 patients had CMR (11%), 7 had PMR (19%), 9 had SD (24%), and 17 had PMD (46%). Grouping responders versus non-responders resulted in sensitivity of 82.1% and specificity of 66.7% to predict PFS < 1 year. PFS was significantly longer (*p* = 0.001) in responders (median PFS not reached days) than in non-responders (median PFS 136 ± 18 days), with HR of 4.93 (1.83–13.30). Grouping non-progressors versus progressors resulted in best performances, with sensitivity of 60.7% and specificity of 100%. PFS was significantly longer (*p* < 0.001) in non-progressors (median PFS 754 ± 399 days) than in progressors (median PFS 109 ± 30 days), with HR of 5.49 (2.26–13.16).

Grouping responders versus non-responders resulted in best performances to predict OS < 2 years, with sensitivity of 82.1% and specificity of 66.7%. OS was significantly longer (*p* = 0.014) in responders (median OS 797 ± 222 days) than in non-responders (284 ± 81 days), with HR of 2.96 (1.20–7.29). Grouping non-progressors versus progressors resulted in sensitivity of 57.1% and specificity of 88.9%. OS was significantly longer (*p* = 0.008) in non-progressors (median OS 460 ± 144 days) than in progressors (190 ± 93 days), with HR of 2.56 (1.25–5.25).

### 2.5. Pseudoprogression and Immune-Related Adverse Effects

**Figure 3:**
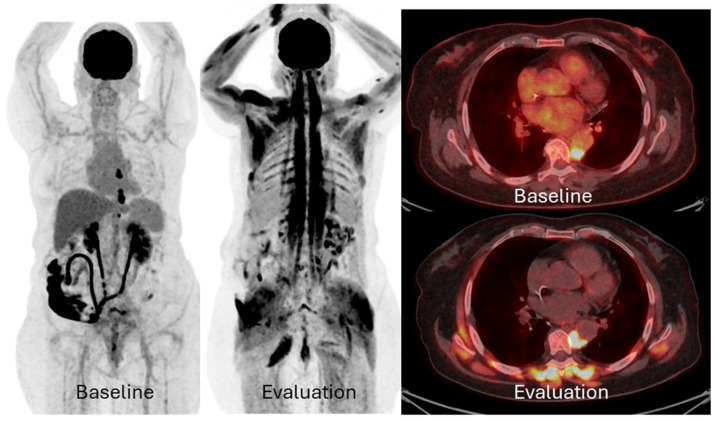
MIP and PET/CT fused images of a patient at baseline and follow-up. She had thoracic peri-aortic metastatic lymphadenopathy that increased in intensity and size between baseline (maximum standard uptake value (SUVmax) 9.7; 18 mm) and evaluation FDG-PET/CT after 3 cycles of Pembrolizumab (SUVmax 14.6; 21 mm). Note that lesion’s FDG uptake on MIP images of evaluation FDG-PET/CT is masked by intense muscular uptake due to immune-related adverse effect of Pembrolizumab that led to adrenal insufficiency. She was classified as progression with PERCIST and imPERCIST5, and as stable disease with PERCIMT. She did not progress until 16 months of Pembrolizumab treatment, and we concluded that she had pseudoprogression. Treatment for adrenal insufficiency was initiated.

Two patients showed a pattern of pseudoprogression (7%), with increased FDG uptake of pre-existing lesions, but PFS > 1 year. Both were classified as PD with PERCIST5, versus one with imPERCIST5 and none with PERCIMT (Figure 3). IrAEs were recorded in three patients (8%), with one case of adrenalitis, one case of hypophysitis, and one case of pneumonitis, all of them being directly or indirectly visible in FDG-PET/CT.

## 3. Discussion

In our study, we assessed the prognostic values on PFS and OS of early response assessment after 2–3 months of Pembrolizumab treatment using the PERCIST5, imPERCIST5, and PERCIMT criteria in metastatic bladder cancer patients. When patients were dichotomized between responders and non-responders, all scores were significantly associated with PFS and OS, with hazard ratios for PFS and OS around 5.0 and 3.0, respectively, meaning that the mean daily probability of clinical progression and death were 5 times and 3 times lower in patients classified as responders versus non-responders, respectively. Sensitivities to predict 1-year PFS and 2-year OS were also satisfying. Considering patients with a metabolic response (CMR or PMR), sensitivity of PERCIST5 and imPERCIST5 were both 85.7% to predict both 1-year PFS and 2-year OS, versus 82.1% for both 1-year PFS and 2-year OS using PERCIMT. This means that patients classified as responders with these scores had around 85% chance of continuing Pembrolizumab without significant clinical progression during the first year after its initiation, as well as 85% chance of not dying within 2 years. By comparison, in the pivotal studies KEYNOTE-045 patients with metastatic urothelial carcinoma who progressed after platinum-based chemotherapy had a 2-year OS of only 16.7% when they were treated with Pembrolizumab [14]. Some of our patients were treated with Enfortumab-Vedotin after they progressed with Pembrolizumab, which could have slightly biased the OS results in our favor, but this does not explain these massive differences in terms of OS, which mostly reflects accurate identification of patients with a good response to immunotherapy. When patients were dichotomized between non-progressors and progressors, all scores were significantly associated with PFS, but only PERCIST5 and PERCIMT were significantly associated with OS. Both imPERCIST5 and PERCIMT are immune-modified response criteria that were initially studied in cohorts of melanoma patients treated with the anti-cytotoxic T-lymphocyte associated protein 4 (CTLA4) agent Ipilimumab. ImPERCIST5 is based on an adaptation to FDG-PET/CT of the morphological score IrRC [15], whereas PERCIMT was retrospectively designed to predict the “clinical benefit” of immunotherapy. When it comes to diagnosing progressive metabolic disease, ImPERCIST emphasizes the evaluation of target lesions, without special considerations for new lesions, whereas PERCIMT only considers new lesions. Of the two opposite approaches, the second worked better in our patients. Indeed, while PERCIMT maintained a good prognostic value on both PFS and OS, it correctly classified the two patients with pseudoprogression features who would experience prolonged clinical benefit from immunotherapy as non-PMD, whereas they were both classified as PMD with PERCIST5. Looking at survival curves, PERCIMT seemed to better discriminate the OS of patients with stable disease versus that of patients with metabolic progression, although the difference was not statistically significant. This is due to the different and somehow more conservative definition of progression used in PERCIMT criteria, in which progression is only diagnosed when 2, 3, or 4 new lesions appear, according to their size. These characteristics make this score clinically useful, as it is simple to use and minimizes the risk of discontinuing an effective treatment. This is especially important in a situation where responses are limited in frequency but can be long-lasting, as is the case with immunotherapy. The original publication of PERCIMT criteria was based on a retrospective analysis of 41 patients treated with Ipilimumab for a metastatic melanoma [11]. In this study, the authors were able to predict the “clinical benefit” (i.e., no-clinical PD versus PD) with a specificity of 100%, as in our study, and a slightly higher sensitivity of 84%. An important difference with our study is that progression rate was lower in their patients (24% vs. 46%), whereas pseudoprogression rate was much higher. Indeed, considering the appearance of ≥1 lesion indicative of PMD would have resulted in the misclassification of 12/21 (57%) of their patients with clinical stable disease. In our study, we observed a lower pseudoprogression rate of 5% using a conservative definition of pseudoprogression, considering this diagnosis only in patients with PFS > 1 year despite being classified as PMD by at least one of the studied criteria. The rate of pseudoprogression can vary between cancer types and immunotherapy treatment, being as high as 15% in melanoma patients treated with the Ipilimumab [15]. Using morphological CT criteria, pseudoprogression rate in patients treated with anti-PD1 or anti-PDL1 has been shown to be lower, around 6.4% for melanoma and around 8% for urothelial carcinoma [16]. In a study of 91 patients with metastatic melanoma treated with ICIs, Ayati et al. also compared the PERCIST5, imPERCIST5, and PERCIMT criteria. They found that the metabolic response with PERCIMT (2.48 versus 1.47 years) and PERCIST (2.57 versus 1.81 years) was significantly associated with longer OS, whereas that of imPERCIST5 was not, which is line with our results [17]. In another study of 76 melanoma patients, PERCIMT was found to be the only score to predict significantly longer survival for metabolic responders versus non-responders after two cycles of immunotherapy, whereas PERCIST was not [18]. In a prospective study of melanoma patients treated mostly with PD-1 inhibitors, Homburg et al. retrospectively studied 29 subjects with suspected pseudoprogression because of an increased uptake of pre-existing lesions or appearance of new lesions on FDG-PET/CT, but without clinical progression [19]. In their study, 34% of patients were ultimately found to have pseudoprogression, while 66% had true progression. Applying PERCIMT criteria had a sensitivity to detect true progression of 37% and a specificity of 80% in these patients. Our results are in line with theirs, showing that PERCIMT criteria can be used to limit the risk of wrongly concluding that immunotherapy is ineffective, at the cost of a reduced sensitivity for detecting true progression. Another study of 67 patients treated with immunotherapy (mostly with Ipilimumab) and evaluated after two and four cycles with FDG-PET/CT was performed by Sachkepedis et al. [18]. They compared the performances of conventional EORTC [20] and PERCIST criteria with those of immunotherapy-modified PERCIMT, imPERCIST5, and iPERCIST criteria [21]. In their study, PERCIMT was the only criteria significantly associated with OS after two cycles of immunotherapy. After four cycles, both conventional and immunotherapy-modified criteria were significantly associated with OS. These good results of PERCIMT criteria have been replicated in a study of 35 patients with advanced non-small cell lung cancer by Castello et al., where they compared morphological RECIST 1.1 [22] and imRECIST [23] criteria, as well as the FDG-PET EORTC, PERCIST, imPERCIST, and PERCIMT criteria after three or four cycles of ICI therapy [24]. All scores were significantly associated with PFS, while only PERCIMT and imPERCIST were associated with OS. Additionally, the prognosis of patients classified as stable disease with CT could be further stratified in terms of PFS and OS using imPERCIST or PERCIMT. A general recommendation is that in case of doubts between progression or pseudoprogression, a confirmatory follow-up FDG-PET/CT study should be performed 4–8 weeks later [10]. This is a practical approach that makes sense, and on which multiple immunotherapy criteria are based. Nevertheless, efforts should be made to reduce the number of patients who need a confirmatory scan to the minimum, without compromising the ability to detect patients who do not respond to immunotherapy and who would benefit from the rapid introduction of a new treatment line. PERCIMT used alone would meet this first specification, but the second one is not completely met. To increase physician’s ability to make the right decision in this setting, one could use predictive factors of response to immunotherapy, or more generally, prognostic factors, be it clinical, biological, histological, or based on imaging. In this regard, baseline metabolic volumes such as metabolic tumor volume (MTV) and total metabolic tumor volume (TMTV) show great promise, with studies identifying them as predictive of response as well as prognostic in patients treated with immunotherapy [25]. In our study, we did not focus on baseline prognostic factors or quantitative metrics such as TMTV, as it will be the subject of another work. Lastly, we detected irAEs in only three patients (8%), which is not enough to assess their prognostic value but was clinically useful.

**Figure 4:**
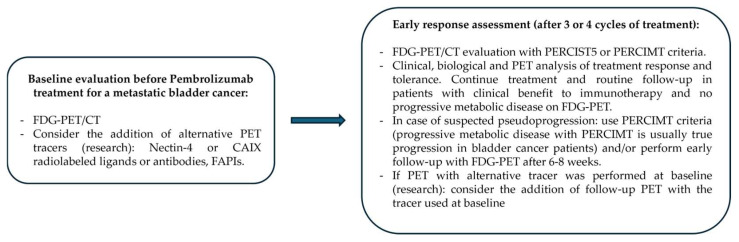
Proposed algorithm for PET follow-up of bladder cancer patients treated with Pembrolizumab. CAIX, Carbonic anhydrase IX. FAPIs, Fibroblast activation protein inhibitors. FDG-PET/CT, [18F]-fluorodeoxyglucose positron emission tomography coupled to computed tomography.

This study has several limitations. Firstly, we used clinical reports as a gold standard for true progression in a retrospective manner, even though FDG-PET/CT results play an important role in oncologists’ decisions to stop or continue immunotherapy. This is probably not a major limitation as FDG-PET/CT were performed in routine follow-up of patients and their reports did not integrate formal response assessment with dedicated criteria. Moreover, oncologists are used to treat patients with immunotherapy beyond imaging progression in our center. Additionally, the prognostic value was good on both PFS and OS in most scenarios in our cohort. Secondly, there were five patients with no valid lesion to use as a baseline target with PERCIST5 or imPERCIST5 that were excluded from the study, leaving only a limited number of patients for analyses and therefore limiting statistical power. We must emphasize here how much simpler it is to use PERCIMT criteria than PERCIST5 or imPERCIST5, as it is applicable to all patients and does not need uptake values to be measured. Additionally, excluding these five patients ensured that all patients had significantly active disease, and increased the homogeneity of the cohort, especially in terms of prognosis. Thirdly, the proportional hazards assumption hypothesis was not verified for any score in our study, as is often the cases in patients receiving immunotherapy, because of a “long-term survivor” effect. This is very unlikely to have biased comparisons of scores, but it limits the generalizability of our HR estimates. Fourthly, in the coming era where total-lesions automated measurements can be performed, it would be important to study how they compare with response criteria in terms of prognostic value, and if they can be combined to better assess patient’s outcome. In that regard, a score such as PERCIMT with an emphasis on new lesions could be well complemented with TMTV measurements, which does not discriminate new from pre-existing lesions but offers a robust estimate of how the global tumor load changed, but that remains to be studied. Fifthly, our analyses and the statistical study of subtle differences between scores performances are limited by the relatively low number of patients in our cohort. Additionally, we did not study the inter-reader reproducibility of the different criteria, as there was only one PET reader in our study. Nevertheless, this is the first study on the subject. Our results should be confirmed in a prospective manner, with a larger number of patients, and multiple PET readers. Lastly, the therapeutical landscape of metastatic bladder cancer treatment is evolving rapidly, with multiple molecules targeting different biological targets that can be overexpressed in bladder cancer being studied or already available in some countries. The most striking example of this came with the results of the EV-302/KEYNOTE 39A, a phase III trial that showed the vast superiority of the Enfortumab-Vedotin antibody–drug conjugate associated with Pembrolizumab versus chemotherapy in the first-line treatment of advanced unresectable or metastatic urothelial carcinomas [26]. This combination of treatment is now considered the standard of care for patients deemed fit for this combination therapy. The FDG-PET/CT evaluation of these patients may share common features with our patients treated with Pembrolizumab alone, but again, this remains to be investigated. Moreover, the diagnostic imaging landscape of bladder cancer is also evolving rapidly. An excellent review by Mc Donald et al. [27] has recently been published on the subject. It provides a state-of-the-art analysis of novel radiopharmaceuticals that are currently being studied for bladder cancer. Briefly, alternate metabolic tracers to FDG, such as ^11^C-acetate, ^11^C-choline, or ^11^C-methionine, are not suitable for bladder cancer imaging, as they showed no significant advantage compared to FDG in this setting [28,29,30]. On the contrary, radiopharmaceuticals targeting biomarkers that are frequently overexpressed in urothelial cancers, such as Nectin-4 [31,32], anhydrase carbonic IX (CAIX) [33,34], urokinase plasminogen activator receptor (uPAR) [35], or trophoblast cell surface antigen 2 (Trop-2) [36], hold great promise for imaging bladder cancer with enhanced contrast and higher specificity. Either ^89^Zirconium labelled antibody or smaller molecules labeled with ^18^Fluor or ^68^Gallium with faster tumor uptake and blood clearance are already being studied for these targets, successfully so far. As for radiopharmaceuticals already available in other clinical settings, prostate specific membrane antigen (PSMA) ligand ^68^Ga-PSMA-11 was tested in a small pilot study of 10 patients with upper tract urothelial carcinoma and shown to be clearly inferior to FDG. PSMA ligands thus holds no promise for bladder cancer imaging [37]. On the other hand, fibroblast activation protein inhibitors (FAPIs) radiopharmaceuticals have demonstrated good results in a range of tumors and appear promising in urothelial cancer [38]. Moreover, cancer-associated fibroblasts (CAFs), the target cells of FAPIs, are linked to tumor ability to evade immune response and FAPIs may thus be especially relevant for the evaluation of patients treated with immunotherapy. In a study of 20 patients treated with preoperative radio-immunotherapy for a locally advanced rectal cancer, the ^68^Ga-FAPI-04 PET/MRI and ^18^FDG PET/CT performances to predict complete pathological response were compared. In this setting, the best results were obtained using the FAPI-PET lean body mass standard uptake peak percentage change (ΔSULpeak), with a sensitivity of 77.8%, a specificity of 100%, and an AUC of 0.93 for a cut-off value set at −63.9%. By comparison, the best metrics for FDG had a sensitivity of 89%, a specificity of 82%, and AUC of 0.80 [39]. FAPI can also be labelled with ^177^Lutethium and used as radioligand therapy. In this setting, synergistic effects of a combination of FAPI radioligand therapy and anti-PDL-1 antibody were recently proven in mice [40]. Thus, promising alternatives to FDG are under investigation for imaging urothelial cancers, including FAPIs and Nectin-4 ligands. In the meantime, we can say that FDG-PET/CT performances for the early assessment of Pembrolizumab response is satisfactory using both PERCIST5 and PERCIMT criteria. A proposed algorithm to be used in the follow-up of bladder cancer patients treated with Pembrolizumab is shown in Figure 4.

## 4. Materials and Methods

### 4.1. Patient Selection and Follow-Up

The patients were selected using the software Chimio^®^ version 2.5 (Computer engineering, Paris, France) to find every patient who was treated with Pembrolizumab for a bladder cancer in Foch hospital (Suresnes, France) from 1 January 2017 to 31 December 2021. The imaging database of the institution was checked for FDG-PET/CT examination performed in the nuclear medicine department. Patients were included in the study if they had both a baseline FDG-PET/CT and evaluation FDG-PET/CT performed after 3 or 4 cycles of Pembrolizumab monotherapy in our center. Exclusion criteria included baseline FDG-PET/CT performed more than 2 months before the start of treatment to ensure that it was representative of metastatic burden at the initiation of Pembrolizumab, a concomitant metastatic solid cancer or hematologic malignancy, and the absence of any valid target lesion for PERCIST or imPERCIST assessment on PET baseline examination.

Medical records of patients were checked for sex, ECOG performance status scale, weight, body max index (BMI), and number of previous line(s) of systemic treatment for bladder cancer. The follow-up of patient was based on oncologist reports until 31 December 2023, including dates of Pembrolizumab start, progression, and death or last follow-up. The date of disease-specific progression was usually the date of last consultation before a new line of treatment was started or when the patient was placed on palliative care. Histological specimen of tumors (primitive lesion and/or metastases) were re-examined whenever available, with assessment of PDL-1 status by TPS and CPS. This retrospective study on data was approved by the local ethic board on 4 May 2022 (registration number IRB00012437). In accordance with French legislation, and more specifically with the French Data Protection Authority (CNIL), research on data, or otherwise known as research not involving the human person, does not require consent. For this “internal study” (French definition of a study conducted using data collected as part of the individual follow-up of patients by the personnel providing this follow-up and for their exclusive use) do not require CNIL authorization. Patients were informed of their general data protection regulation (GDPR) rights, notably the opportunity to object to the reuse of their personal data for research purposes. The database was pseudonymous in accordance with the law. A copy of the information available to patients is presented in Supplementary Materials.

### 4.2. FDG-PET/CT Protocol

Patients were instructed to fast for a minimum of 6 h and to avoid physical strenuous activity for at least 24 h prior to the scan. Blood glucose level was measured and was ≤11 mmol/L in all patients. An activity of 3–4 MBq/kg of FDG was injected and patients were left to rest on a chair. FDG-PET/CT acquisition was started just after bladder voiding at 60 +/− 10 min post-injection. It was performed from vertex to mid-thigh, in supine position, on an integrated Discovery 610 Elite PET/CT system (GE Healthcare^®^, Chicago, IL, USA) before August 2019, and on a Discovery MI 5 digital PET/CT system (GE Healthcare^®^) thereafter. The most recent PET/CT system was calibrated to give the same SUV values as the older one when it was installed. An iterative reconstruction with 32 subgroups and 2 iterations was used in the first case, and a Bayesian penalized-likelihood reconstruction algorithm (Q.clear) with a β parameter at 600 in the second. A matrix of 256 × 256 was used in both cases. Parameters for CT images were 100 or 120 kV according to patient BMI, 180 mAs or 100–300 mAS with tube current modulation depending on which camera was used, slice thickness 2.5 mm, pitch 1.3, and matrix 512 × 512. No CT contrast agent was used.

### 4.3. FDG-PET/CT Analysis

PET/CT images were interpreted by a nuclear medicine physician blinded to patient medical data using Syngo.Via VB 50 (Siemens ^®^, Erlangen, Germany) software. Response was assessed using both PERCIST 1.0, imPERCIST5, and PERCIMT criteria. For PERCIST 1.0, we used the variation of up to five target lesions (PERCIST5). Briefly, the standard uptake value normalized to lean body mass peak value (SULpeak) of the five most hypermetabolic lesions (maximum two lesions by organ) were measured at baseline and evaluation, and their sum was recorded. Lesion’s SULpeak had to be above 1.5 × healthy liver mean SUL (SULmean) to be considered a valid target. Patient’s response was classified as PMD when there was an increase > 30% in SULpeak sum and/or a visible increase in extent of FDG tumor uptake and/or new FDG-avid tumor lesions. In the absence of PD features, they were classified as having CMR when there was complete resolution of uptake in target lesions, PMR when there was a reduction of least 30% in SULpeak of target lesions sum, and SD in any other case.

Response assessment with imPERCIST5 was performed in the same way as described for PERCIST 1.0, except that the appearance of new lesions alone did not result in PMD. Thus, PMD was defined only by an increase in the sum of SULpeak by 30%. New lesions were included in the sum of SULpeak if they showed higher uptake than existing target lesions or if fewer than 5 target lesions were detected on the baseline scan, with a limit of maximum two lesions by organ.

With PERCIMT criteria, PMD was defined as the appearance of ≥4 new hypermetabolic lesions with functional diameter of less than 1.0 cm, or ≥3 new lesions of more than 1.0 cm in functional diameter, or ≥2 new lesions of more than 1.5 cm in functional diameter. In the absence of PMD features, patients were classified as having CMR when there was complete resolution of all pre-existing FDG avid lesions, as having PMR when there was complete resolution of some pre-existing lesions, and SD in any other case.

### 4.4. Histological Analysis

Most recent histological specimens available (primitive tumors or metastases) were reassessed to classify tumors as classic urothelial carcinoma, variant urothelial carcinoma, or non-urothelial carcinoma bladder cancers. A quantification of the PD-L1 expression was performed by immunochemistry using CPS and TPS. Briefly, TPS represents the percentage of viable tumor cells with PD-L1 membrane staining, while CPS includes PD-L1-positive tumor and immune cells relative to total tumor cells. 

### 4.5. Statistical Analysis

Sensitivity and specificity of the scores to predict PFS < 1 year and OS < 2 years were assessed in ROC curve analysis, as they were considered clinically relevant thresholds. Best performances were considered to be those that maximized the sum of sensitivity + specificity. A patient response classified as PMD with at least one score was considered to have pseudoprogression when its PFS > 1 year. Prognostic value on PFS and OS of the response assessment scores were analyzed with Cox regression model, with calculation of hazard ratios. The proportional hazards assumption was tested with the interaction term method. Kaplan–Meier survival curves were compared with the log-rank test. A *p*-value < 0.05 was considered statistically significant, without adjusting for multiple comparison for these exploratory analyses. Median or mean (standard deviation) values are given depending on the variable of interest. The statistical analyses were performed with IBM SPSS^®^ statistics version 26 software (Armonk, New York, NY, USA).

## 5. Conclusions

FDG-PET/CT is a valuable tool for early assessment of response in metastatic bladder cancer patients treated with Pembrolizumab. PERCIMT criteria is simple to use and correctly identified patients with pseudoprogression in our cohort. 

## Figures and Tables

**Table 1:** Patient characteristics.

	Median or N	Mean (±Standard Deviation) [Range] or %
Sex	Male: 26 Female: 11	70% 30%
Age (years)	72	69.7 (±10.1) [33–82]
Weight (kg)	72.5	73 (±15.0) [49–118]
BMI	25.1	25.5 (±4.8) [17.9–39.0]
ECOG PS	9: 11 22: 25 6: 6 3 or more: 0	24% 60% 16% 0%
Previous lines of treatment	1: 25 2: 10 3: 2	68% 27% 5%
Histology	Classic UC: 26 Variant UC: 6 Non-UC histology: 1	79% 18% 3%
PDL-1 TPS	5% <1%: 11	18.3 (±29)% [0–90%] 44%
PDL-1 CPS	10% <1%: 9	21.2 (±29)% [0–90%] 36%
Cycles received	8	11.6 (± 11.7) [3–47]
PFS (days)	152 (±29)	361 (±70) [52–1233]
OS (days)	363 (±47)	517 (±70) [68–1384]

BMI, body mass index. ECOG PS, Eastern Cooperative Oncology Group performance status. CPS, combined positive score. OS, overall survival. PDL-1, programmed cell death ligand. PFS, progression-free survival. TPS, tumor proportion score. UC, urothelial carcinoma.

**Table 2:** Hazard ratios of response assessment criteria.

Responders Versus Non-Responders	HR for PFS (95%CI)	HR for OS (95%CI)
PERCIST5	5.6 (1.9–16.5)	3.1 (1.2–8.2)
ImPERCIST5	5.4 (1.8–15.9)	3.1 (1.2–8.1)
PERCIMT	4.9 (1.8–13.3)	3.0 (1.2–7.3)
Non-progressors versus progressors		
PERCIST5	5.5 (2.3–13.3)	2.2 (1.0–4.8)
ImPERCIST5	3.7 (1.7–8.3)	1.6 (0.8–3.3)
PERCIMT	5.5 (2.3–13.2)	2.6 (1.2–5.2)

HR, hazard ratio. 95%CI, 95% confidence interval.

## Data Availability

The original contributions presented in this study are included in the article/Supplementary Material. Further inquiries can be directed to the corresponding author.

## Supplementary Materials

The following supporting information can be downloaded at: https://www.mdpi.com/article/10.3390/ph18050701/s1.
