# Supplementary material for: Comparison of PERCIST5, imPERCIST5, and PERCIMT Criteria for Early Assessment of Pembrolizumab Response with FDG-PET/CT in Metastatic Bladder Cancer Patients
Source: Pharmaceuticals (Basel). 2025 May 9;18(5):701. doi: 10.3390/ph18050701 (PMC12115037; doi:10.3390/ph18050701)
Supplement: Supplementary file 1 [file pharmaceuticals-18-00701-s001.zip › pharmaceuticals-3500581-supplementary.pdf]

Supplementary materials

S1 : Non-opposition form

"Vous êtes suivi à l'hôpital Foch, établissement de santé privé d'intérêt collectif, qui a pour missions le soin, l'enseignement et la recherche. Vos données médicales recueillies lors de votre prise en charge (consultation, hospitalisation, chirurgie, échantillons biologiques, imagerie, etc.) peuvent donc être réutilisées, sauf opposition de votre part, à des fins de recherche dans le domaine de la santé, sous la responsabilité de l'hôpital Foch. Depuis 2020, l'Hôpital est doté d'un Entrepôt de Données de Santé (EDS) afin d'accélérer la recherche et de faire progresser la médecine actuelle vers la médecine du futur. Pour plus de renseignement sur la recherche à l'hôpital Foch vous pouvez consulter notre site internet : https://www.hopital-foch.com/patients-familles/recherche/ Pour vous opposer à l'utilisation de vos données personnelles à des fins de recherche, vous pouvez vous adresser au délégué à la protection de donnée de l'hôpital Foch en le contactant par mail à dpo@hopital-foch.com ou à l'adresse postale suivante : Délégué à la protection des données de l'Hôpital Foch Hôpital Foch 40, rue Worth BP 36 - 92151 Suresnes."

S2 : English translation of the non-opposition form

"You are being treated at Foch Hospital, a private non-profit healthcare institution dedicated to patient care, teaching, and research. Medical data collected during your treatment (consultations, hospitalizations, surgeries, biological samples, imaging, etc.) may therefore be reused, unless you object, for health research purposes under the responsibility of Foch Hospital. Since 2020, the hospital has implemented a Health Data Warehouse (EDS) to accelerate research and advance modern medicine toward the medicine of the future.For more information about research at Foch Hospital, you can visit our website: https://www.hopital-foch.com/patients-familles/recherche/. To object to the use of your personal data for research purposes, you may contact the Data Protection Officer (DPO) of Foch Hospital by email at dpo@hopital-foch.com or at the following postal address: Data Protection Officer of Foch Hospital, Hôpital Foch, 40, rue Worth BP 36, 92151 Suresnes, France."
